# Incidence and risk factors for postoperative nosocomial pneumonia in elderly patients with hip fractures: A single-center study

**DOI:** 10.3389/fsurg.2023.1036344

**Published:** 2023-02-07

**Authors:** Xiao Tong, Caizhe Ci, Jia Chen, Minghong Sun, Hongbo Zhao, Haiqiang Wei, Tieqiang Yu, Hui Wang, Weixin Yang

**Affiliations:** ^1^ Department of Orthopaedic, The First Hospital of Hebei Medical University, Shijiazhuang, China; ^2^ Department of Orthopaedic, The Third Hospital of Shijiazhuang City, Shijiazhuang, China; ^3^ Internal Medicine-Cardiovascular Department, The Third Hospital of Hebei Medical University, Shijiazhuang, China; ^4^ Department of Orthopaedic, Hebei General Hospital, Shijiazhuang, China; ^5^ Department of Orthopaedic, The Second Hospital of Tangshan, Tangshan, China

**Keywords:** hip fracture, clinical epidemiology, influence, risk factors, geriatric population, nosocomial pneumonia

## Abstract

**Objective:**

Postoperative nosocomial pneumonia is a terrible complication, especially for elderly patients. This study attempts to investigate the incidence and risk factors for postoperative nosocomial pneumonia and its influence on hospitalization stay in elderly patients with hip fractures.

**Methods:**

This study retrospectively retrieved hospitalization records of patients who presented a hip fracture and underwent surgeries in our institution between January 2014 and December 2021. Postoperative new-onset pneumonia was determined in accordance with discharge diagnosis. Multivariate logistic regression analysis was performed to identify the associated risk factors with pneumonia, and its influence on total hospitalization stay or postoperative hospitalization stay was investigated by multivariate linear regression analyses.

**Results:**

Totally, 808 patients were included, among whom 54 developed a pneumonia representing the incidence rate of 6.7% (95% CI, 5.0%–8.4%). Six factors were identified as independently associated with pneumonia, including advanced age (OR, 1.50 for each 10-year increment), history of chronic respiratory disease (OR, 4.61), preoperative DVT (OR, 3.51), preoperative delay to operation (OR, 1.07 for each day), surgical duration ≥120 min (OR, 4.03) and arthroplasty procedure (OR, 4,39). When adjusted for above confounders, pneumonia was significantly positively associated with total hospitalization stay (standardized coefficient, 0.110; *p* < 0.001) and postoperative hospitalization stay (standardized coefficient, 0.139; *p* < 0.001).

**Conclusions:**

This study identified multiple factors associated with postoperative pneumonia and its influence on prolonging hospitalization stay, which would facilitate preventive targeted intervention into implementation for individuals with different risk profiles.

## Introduction

Surgical treatment, *via* either arthroplasty or osteosynthesis, has been well established as the gold standard for management of hip fracture in elderly patients, who are generally frail and comorbid (1). This strategy allows early mobility and initiation of postoperative exercises, and thus helps to prevent or reduce many complications that often occur after conservative treatments, e.g., deep venous thrombosis (DVT) of lower extremities, neuromuscular dysfunction or even mortality within early period (2, 3). Despite that, postoperative nosocomial pneumonia, which would cause systemic dysfunction and even death, is prevalent in 4.7%–16.3% of elderly hip fracture patients (4–7). Not only the direct adverse events, but also the great costs from prolonged hospitalization stay and re-admission to hospital constitute a substantial burden for patients and the public health-care systems (4, 8, 9).

From the cost-effective point of view, to prevent is most favorable than to treat. Indeed, during the past decade, researchers have made substantial attempts to address the prevention, and numerous influential factors have been well established, e.g., male sex, advanced age, obesity, history of a chronic respiration disease, active smoking, undernutrition, greater comorbidity index (American Society of Anesthesiologists score ≥ III) or presence of a specific comorbidity or condition (diabetes, renal insufficiency, dementia, anemia, hypoalbuminemia, lower oxygen status), delay to surgery, surgical method (arthroplasty vs. osteosynthesis) and mechanical ventilation (4, 6, 8, 10–12). However, some limitations should be noted, including but not limited to relatively small sample size, inadequate confounders for adjustment, and inaccuracy in data collection. In addition, one may neglect that, up to 35%, of elderly patients with hip fracture had preoperative DVT, despite prophylactic thromboembolic drugs were routinely administered (13). It is possible that the hypercoagulability and the relatively poor venous status associated with DVT in elderly trauma patients might also be contributors for nosocomial pneumonia, however, this has not been investigated in literature. Furthermore, in China, for seeking better surgical treatment, patients generally are transferred or admitted to higher-level tertiary referral hospitals, easily leading to centralization of hip fracture surgery and the delay to surgery, which is a well-known risk factor for many complications, even mortality.

Given the above, we performed this study, with aims to investigate the incidence and risk factors associated with postoperative nosocomial pneumonia in elderly patients with hip fractures, and its influence on hospitalization stay, a direct factor related to the total health care costs.

## Materials and methods

The study was performed in accordance with the Declaration of Helsinki and the study protocol was approved by the ethics committee of the Second Hospital of Tangshan prior to its commencement, which waived the requirement for informed consent due to the retrospective nature.

We reviewed patients who presented with and underwent a surgery for an acute hip fracture between January 2018 and December 2021 in the Second Hospital of Tangshan, an 800-bed orthopaedics-specialized hospital serving a population of 7.7 million people. The inclusion criteria were age of 60 years or older, diagnosis of hip fracture (femoral neck or intertrochanteric) definitely surgically treated and complete medical records. The exclusion criteria were injury mechanism of high-energy impact (fall from a height, traffic accident or others), subtrochanteric fracture, open fracture, pathological fractures, polytrauma or concurrent fractures, non-operative treatment or delay to operation >21 days after fracture, history of any operation on the affected hip, malignancies, presence of preoperative pneumonia, long-term use of glucocorticoid or missing information on variables of interest.

### Definition and identification of pneumonia

Two researchers (X Tong and C Ci) were responsible for data exaction, *via* review of the hospital’ electronic database. Postoperative nosocomial pneumonia was defined as a postoperative pneumonia occurring ≥48 h after hospital admission, which was documented in the discharge abstract. It was diagnosed in accordance with the Guidelines (14), on basis of following criteria: (1) typical clinical presentations and physical examination findings, showing cough, expectoration, fever or hypothermia (body temperature >38 °C or body temperature < 36 °C), chest pain, moist rale on lung auscultation or lung consolidation signs; (2) blood tests showing increase or decrease of number of white blood cell (WBC) (>10 * 10^9^/L or white cell count <4 * 10^9^/L) and the percentage of neutrophils; (3) Chest x-ray or CT scanning showing signs of pneumonia; and 4, blood or sputum culture revealing the same causative pathogens for two consecutive times.

### Variables of interest

Demographics features and potential risk factors were extracted from the records by the same investigators (X Tong and C Ci). These variables included sex, age, height and weight and the calculated body mass index (BMI), lifestyles (active smoking, alcohol drinking), comorbidities or conditions (hypertension, diabetes, chronic respiratory disease, heart disease, cerebrovascular disease, liver disease, renal disease, presence of preoperative DVT), fracture location (femoral neck or intertrochanteric), surgery-related data (delay to operation after fracture, anesthesia pattern, surgical duration, American Society of Anesthesiologists (ASA) score, intraoperative bleeding, allogeneic blood transfusion and operative procedure (arthroplasty or osteosynthesis). In addition, some blood test indexes immediately after admission were also extracted, including serum albumin level, albumin/globulin ration, WBC count, neutrophil count, lymphocyte count, red blood cell (RBC), hemoglobin, hematocrit, hypersensitivity C-reactive protein (HCRP), lactate dehydrogenase (LDH), creatinine and fasting blood glucose (FBG).

In accordance with the criteria proposed specifically for Chinese adults (15), obesity was defined as BMI ≥28 kg/m^2^. Active smoking or alcohol drinking was defined as regular consumption of cigarettes or alcohol within 6 months before the index operation (16). Preoperative DVT was diagnosed by duplex ultrasonography or venography, which was a routine procedure for patients with hip fracture before surgery. Comorbidities or conditions were self-reported by patients after admission and documented by the initial clinicians on-duty. The blood test indexes were categorized according to the manufacturer-recommended reference ranges.

### Statistical analysis

Continuous variables were presented with mean and standard deviation (SD), and were explored for their normality distribution status by Kolmogorov–Smirnov test; and the difference between patients with and without pneumonia was detected by Student-*t* test for normally distributed data or by Mann Whitney-*U* test for skewedly distributed data. Categorical variables were presented as number and percentage, and the between-group difference was detected by Chi-square or Fisher exact test, as appropriate.

The incidence rate of postoperative nosocomial pneumonia was calculated by dividing the total number of patients by the number of those who developed pneumonia during hospitalization stay. Variables that tested with *P* values <0.10 in the above univariate analyses were further entered into the multivariate logistic regression analysis to identify their potential independent effect on incidence of postoperative nosocomial pneumonia. During this procedure, the stepwise backward mode was applied to eliminate the less associated factors, and those with *P* value <0.10 were retained in the final model. The magnitude of association was indicated by the odd ratio (OR) and its 95% confidence interval (95% CI). To evaluate the goodness-of-fit of the final model, Hosmer–Lemeshow test was applied with *P *> 0.05 indicating the acceptable result; also, adjusted Nagelkerke *R*^2^ value was used to quantify the magnitude of goodness-of-fit, with <0.750 deemed as acceptable result, with lower value suggesting a better model fit.

For investigation of effect of pneumonia on the hospitalization stay, we performed the multiple linear regression, with hospitalization stay in days as outcome variable and pneumonia as independent variables and above-mentioned variables tested with *p* value <0.10 as co-variables for adjustment. The “enter” mode was applied. The collinearity between independent variables was examined by variance inflation factor (VIF), with VIF ≥ 3 suggestive of multicollinearity and the related factors were not included. Regression coefficient (B) with 95% CI and the standard regression coefficient (Beta) were used to indicate the association magnitude.

For all analyses, *P *< 0.05 was considered as significant. All statistical analyses were performed by SPSS25.0 package (IBM, Armonk, NY, United States).

## Results

**Figure 1:**
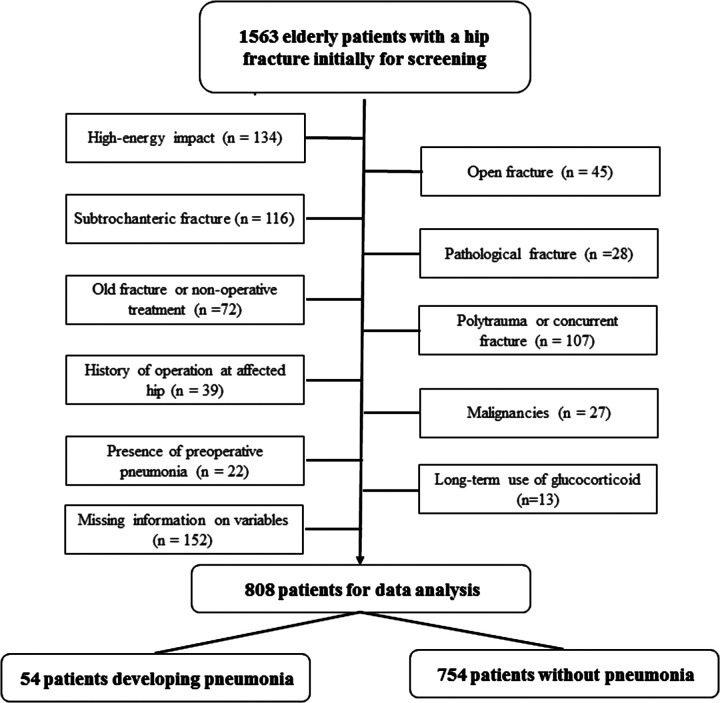
The flowchart showing the screening of eligible participants.

Within the study period, there were 1,563 elderly hip fractures treated in our institution, and 755 were excluded due to various reasons, e.g., high-energy impact (134), subtrochanteric fracture (116), open fracture (45), pathological fractures (28), polytrauma or concurrent fractures (107), non-operative treatment or delay to operation >21 days (72), history of any operation on the affected hip (39), malignancies (27), presence of preoperative pneumonia (22), long-term use of glucocorticoid (13) or missing information on variables of interest (152). This left 808 eligible patients for data analysis (Figure 1).

Fifty-four patients developed pneumonia after operation, indicating an incidence rate of 6.7% (95% CI, 5.0%–8.4%). Compare to those without developing pneumonia, patients who had pneumonia had an older age (76.9 ± 8.2 vs. 72.7 ± 8.6; 38.9% vs. 24.8% for age ≥80 years), higher prevalence rate of hypertension (61.1% vs. 46.0%), diabetes (33.3% vs. 20.7%), respiratory disease (13.0% vs. 3.2%), presence of preoperative DVT of bilateral extremities (29.6% vs. 13.7%) and a longer preoperative waiting (7.2 ± 4.7 vs. 5.4 ± 3.4 days; 46.3% vs. 27.7% for preoperative waiting over 7 days), needed a longer surgical duration (131.7 ± 40.0 vs. 112.7 ± 41.8 min; 79.6% vs. 47.2% for procedure lasting over 120 min), a higher proportion of arthroplasty procedure (75.9% vs. 54.9%) and a higher proportion of elevated WBC (38.9% vs. 25.5%) (Table 1). Two (3.7%) patients in pneumonia group and 14 (1.9%) in non-pneumonia group died during the index hospitalization, without significant difference (*P* = 0.290).

**Table 1:** Univariate analysis of variables between pneumonia and non-pneumonia patients.

Variables	Pneumonia (*n* = 54)	Non-Pneumonia (*n* = 754)	*P*
	Mean ± SD or count (%)	Mean ± SD or count (%)	
**Sex (males)**	19 (35.2)	280 (37.1)	0.774
**Age**	76.9 ± 8.2	72.7 ± 8.6	<0.001
≥80 years	21 (38.9)	187 (24.8)	0.022
**BMI (**kg/m^2^**)**	23.3 ± 3.8	23.6 ± 3.7	0.600
Obesity	8 (14.8)	78 (10.3)	0.304
**Hypertension**	33 (61.1)	347 (46.0)	0.032
**Diabetes mellitus**	18 (33.3)	156 (20.7)	0.029
**Respiratory disease**	7 (13.0)	24 (3.2)	<0.001
**Heart disease**	15 (27.8)	183 (24.3)	0.563
**Cerebrovascular disease**	15 (27.8)	212 (28.1)	0.957
**Liver disease**	3 (5.6)	18 (2.4)	0.332
**Renal disease**	6 (11.1)	53 (7.0)	0.399
**Preoperative DVT**	16 (29.6)	103 (13.7)	<0.001
**Cigarette smoking**	11 (20.4)	129 (17.1)	0.541
**Alcohol drinking**	18 (33.3)	236 (31.3)	0.756
**Fracture location**			0.089
Femoral neck	43 (79.6)	517 (68.6)	
Intertrochanteric	11 (20.4)	237 (31.4)	
**Preoperative stay (days)**	7.2 ± 4.7	5.4 ± 3.4	<0.001
**≥7d**	25 (46.3)	209 (27.7)	0.004
**Hospital stay (days)**	20.3 ± 10.3	14.1 ± 6.6	<0.001
**Intraoperative bleeding (ml)**	179.6 ± 340.0	109.6 ± 255.6	0.058
**Intraoperative blood transfusion**	14 (25.9)	152 (20.2)	0.311
**Surgical duration (min)**	131.7 ± 40.0	112.7 ± 41.8	<0.001
**≥120**	43 (79.6)	356 (47.2)	<0.001
**Procedure**			0.003
Arthroplasty	41 (75.9)	414 (54.9)	
Osteosynthesis	13 (24.1)	340 (45.1)	
**ASA**			0.083
I-II	28 (51.9)	480 (63.7)	
III-IV	26 (48.1)	274 (36.3)	
**Anesthesia (general)**	34 (63.0)	450 (59.7)	0.635
**Albumin (<35 g/L)**	30 (55.6)	425 (56.4)	0.908
**A/G**			0.284
1.2–2.4	37 (68.5)	587 (77.9)	
<1.2	17 (31.5)	167 (22.1)	
**HCRP (>8 mg/L)**	43 (79.6)	624 (82.8)	0.558
**LDH (>250 U/L)**	21 (38.9)	226 (30.0)	0.170
**Sodium concentration (<135 mmol/L)**	18 (33.3)	309 (41.0)	0.269
**FBG (>6.1 mmol/L)**	28 (51.9)	371 (49.2)	0.707
**Creatinine (>111 *μ*mol/L)**	8 (14.8)	65 (8.6)	0.198
WBC (>10 * 10^9^/L)	21 (38.9)	192 (25.5)	0.031
Neutrophil count (>6.3 * 10^9^/L)	29 (53.7)	384 (50.9)	0.693
Lymphocyte count (<1.1 * 10^9^/L)	29 (53.7)	371 (49.2)	0.523
^#^ **RBC (<Lower limit)**	22 (40.7)	376 (49.9)	0.195
^#^ **Hemoglobin (<Lower limit)**	20 (37.0)	367 (48.7)	0.098
^#^ **Hematocrit (<Lower limit)**	32 (59.3)	519 (68.8)	0.145
Platelet count (>300 * 10^9^/L)	9 (16.7)	85 (11.3)	0.232

SD, standard deviation; BMI, body mass index; ASA, american society of anesthesiologists; WBC, white blood cell; A/G, albumin/globulin; HCRP, hypersensitive c-reactive protein; LDH, lactate dehydrogenase; FBG, fasting blood glucose; RBC, red blood cell.

^#^ Reference range was applied stratified by sex: RBC: Female, 3.5–5.0 * 10^12^/L and males, 4.0–5.5 * 10^12^/L; Hemoglobin: Females, 110–150 g/L and males, 120–160 g/L; Hematocrit: Females, 35%–45%; males, 40%–50%.

In the multivariate logistics regression analysis, age (OR,1.50% and 95% CI, 1.10–1.92 for each 10-year increment), history of chronic respiratory disease (OR, 4.61; 95% CI, 1.70–12.54), preoperative DVT (OR, 3.51; 95% CI, 1.74–10.48), preoperative waiting (OR, 1.07; 95% CI, 1.01–1.15 for each day increment), surgical duration ≥120 min (OR, 4.03; 95% CI, 1.96–8.27) and arthroplasty procedure (OR, 4,39; 95% CI, 1.87–10.31) (Table 2). The Hosmer–Lemeshow test showed the good fitness (*X*^2 ^= 6.328, *P* = 0.556, Nagelkerke *R*^2^ = 0.197).

**Table 2:** Multivariate analysis of factors associated with postoperative nosocomial pneumonia in patients with a hip fracture.

Variables	OR	95% CI		*P*
		Lower limit	Upper limit	
Age (each 10-year increment)	1.50	1.10	1.92	0.011
Diabetes	1.77	0.91	3.43	0.092
Chronic respiratory disease	4.61	1.70	12.54	0.003
Presence of preoperative DVT	3.51	1.74	10.48	0.001
Preoperative waiting (in each day increment)	1.07	1.01	1.15	0.039
Surgical duration >120 min	4.03	1.96	8.27	<0.001
Procedure (arthroplasty vs. osteosynthesis)	4.39	1.87	10.31	0.001

The total hospitalization stay was 20.3 ± 10.3 days in patients developing pneumonia, significantly longer than that (14.1 ± 6.6) in those without (*P *< 0.001). The postoperative hospitalization stays (calculated by subtracting the preoperative stay from the total hospitalization stay) was significantly longer in patients developing pneumonia than those without (13.1 ± 8.9 vs. 8.8 ± 5.0 days, *P *< 0.001) (Table 1). The multivariate linear regression analyses showed pneumonia was significantly positively with the total hospitalization stay (B, 3.11; 95% CI, 1.67–4.84; Beta, 0.110; *P *< 0.001) and the postoperative hospitalization stay (B, 3.93; 95% CI, 2.35–5.49; Beta, 0.139; *P *< 0.001) (Tables 3, 4). The VIFs were ranging 1.034–2.923, and 1.036–3.088, respectively; indicating no multicollinearity for any factor.

**Table 3:** ^&^Multivariate linear regression analysis showing pneumonia significantly positively associated with total hospitalization stay, together with other 3 factors.

Variables	*B*	95% CI	Beta	*T*	*P*
Pneumonia	3.119	1.672 to 4.849	0.110	3.986	<0.001
Preoperative stay (day)	1.101	0.988 to 1.123	0.549	19.223	<0.001
Surgical duration ≥120 min	1.820	1.040 to 2.600	0.129	4.580	<0.001
Procedure (osteosynthesis vs. arthroplasty)	2.205	1.348 to 3.063	0.155	5.047	<0.001

B, unstandardized coefficient; Beta, standardized coefficient indicating the strength of influence; and T, statistic of the regression.

^&^ Covariables included in this multivariate model were pneumonia, age in continuous variable, diabetes, history of respiratory disease, preoperative DVT, surgical duration, procedure pattern.

**Table 4:** ^^^Multivariate linear regression analysis showing pneumonia significantly positively associated with postoperative hospitalization stay, together with other 3 factors.

Variables	*B*	95% CI	Beta	*T*	*P*
Pneumonia	3.934	2.351 to 5.493	0.139	4.104	<0.001
Preoperative stay (day)	0.181	0.075 to 0.287	0.117	3.348	0.001
Surgical duration ≥120 min	1.575	0.837 to 2.313	0.144	4.191	<0.001
Procedure (osteosynthesis vs. arthroplasty)	2.254	1.444 to 3.065	0.205	5.462	<0.001

B, unstandardized coefficient; Beta, standardized coefficient indicating the strength of influence; and T, statistic of the regression.

^^^ Covariables included in this multivariate model were pneumonia, age in continuous variable, diabetes, history of respiratory disease, preoperative DVT, surgical duration, procedure pattern.

## Discussion

In the present study, we found the incidence of postoperative nosocomial pneumonia was 6.7% in elderly patients with a hip fracture and identified 6 independent factors, including elder age, history of chronic respiratory disease, present preoperative DVT of bilateral extremities, prolonged preoperative waiting, surgical duration ≥120 min and arthroplasty procedure. We also identified that pneumonia was positively associated with total hospitalization stay and postoperative hospitalization stay.

The rate of postoperative nosocomial pneumonia of 6.7% in elderly patients with a hip fracture was in range of those reported in literature on this subject, which, however, varied greatly between 4.7% and 16.3% (4–7). The reasons for such variation might be various, primarily from patient selection, treatment pattern (surgery or conservation), wide definitions of pneumonia, study design and sample size. For example, in one study of 418 hip fracture patients aged 60 years or older, Yan et al. (7) reported a highest incidence of 16.3% for postoperative pneumonia. That might be explained by the greater proportion (29.0%, 7-times as ours) of previous respiratory system disease in their population, a substantial risk (OR, 4.61) for pneumonia identified in our study. Another important factor might be that 15.8% of included patients were conservatively treated, also a well-established risk factor for various complications and adverse outcomes, including pneumonia and even morality (3, 17). In contrast, in the study there the lowest incidence rate (4.7%) was reported, the lower proportion of smokers (4.4%, about 1/4 as ours) and relatively low proportion of femoral neck fracture, a substantial proportion of which requires arthroplasty, might contribute greatly (6).

In consistence with previous findings (1, 18), postoperative pneumonia was re-confirmed as a risk factor for prolonged hospitalization stay in this study, and was associated with additional 6.2 day and 4.3 day for total and postoperative hospitalization stay, respectively. Their influence on costs from public health care system and patients is remarkable, which, exactly, underscores the importance of prevention of postoperative pneumonia.

Among 6 factors identified, most have been well established, e.g., elder age (2, 6, 19), history of chronic respiratory disease (20, 21), prolonged preoperative waiting (22, 23) and longer surgical duration (24) and arthroplasty procedure (6). The first two factors related to the patients’ systemic functional decline and the poorer cardiorespiratory reserves, e.g., decline of breathing strength, lung compliance, cough reflex and respiratory defense, providing the basis and intrinsic conditions for pneumonia (25, 26). The prolonged preoperative waiting, on one hand, might reflect the frail systemic conditions, comorbidities or severe injury that require more time to optimize to improve the tolerance to surgery. On the other hand, the physical and psychological changes (e.g., anxiety and sleep disorder) secondary to prolonged preoperative hospitalization stay should also contribute to lowering the resistance to surgical trauma (27), thus increasing the risk of pneumonia. Therefore, for those older, especially aged >80 years, and having chronic respiratory disease, simplification of procedure to admit and multidisciplinary intervention to achieve a fastest medical optimization might be more effective, e.g., setting of dedicated, organized and comprehensive orthogeriatric care wards (28).

Greater surgical trauma meant the increased body inflammatory/immune response, and the prolonged surgical duration and arthroplasty procedure (vs. osteosynthesis), undoubtedly, contributed predominantly to this effect (6, 7). Despite the international guidelines recommending specific surgical procedure for different fracture patterns of hip fractures, taking age and systemic conditions into consideration, but that seemed more applicable for femoral neck fractures. Because, for intertrochanteric fracture, more options were available, including dynamic hip screw, Gamma screw and proximal femoral nail and variants, which possibly caused less-experienced surgeons to be trapped in the dilemma of choose (29). Thus, out results emphasize the importance of thorough understanding and grasping the indications for hip fracture surgery, thereby choosing a simple and fast surgical method to shorten the surgical duration and reduce the risk of intraoperative exposure.

Preoperative DVT of bilateral lower extremities were detected in 6.8%–35% of patients with a hip fracture, even if prophylactic thromboembolic agents are routinely administered (13). However, no studies linked this to the risk of pneumonia. In this study, we got the relatively strong relationship magnitude (OR, 3.51). The underlying mechanism is unclear, but we can obtain some useful information from other studies. Minno et al. (30) conducted a meta-analysis of studies on relationship between COVID-19 and venous thromboembolism (VTE), and found the incidence of VTE was 31.3% in COVID-19 patients far greater than that for general patients, also for hip fracture patients (14.7% in this study). Factors that contribute to developing DVT, such as endothelial injury, venous blood stasis and hypercoagulability, are also potentially playing a role in pneumonia. In addition, platelet activation (a component in DVT) would promote the release of vasoactive mediators and thus increase the pulmonary vascular resistance (31), potentially creating an improved condition for bacterial colonization. Regardless, patients detected with preoperative DVT should be placed more attention on the risk of postoperative pneumonia, and preventive targeted measures to eliminate embolus and enhance respiratory dynamics and muscle function should be considered into practice for this population.

### Strengths and limitations

The strengths of this study included the relatively sample and inclusion of numerous variables for adjustment. However, the potential limitations should also be noted. First, the retrospective design had the intrinsic limitations in data collection, especially that comorbidities or conditions were self-reported by patients. Second, the single-center design might have compromised the representativeness of sample, and our institution was an orthopaedics-specialized hospital, thus further deteriorating the issue of selection bias. Also, the generalizability of these finding might be less applicable to other settings. Third, as with every logistic regression analysis, the unknown, unmeasured or not considered factors make the confounding effects remain. Fourth, due to the observational nature, the findings were associative rather than causative, and therefore should be interpreted with caution.

## Conclusion

Nosocomial pneumonia was prevalent in 6.7% of patients following surgical treatment, and was positively associated with total and postoperative hospitalization stay. Six factors were identified, including elder age, history of chronic respiratory disease, preoperative DVT, prolonged preoperative waiting, surgical duration ≥120 min and arthroplasty procedure. These findings help stratify patients regarding the risk of pneumonia and more importantly, facilitating preventive targeted intervention into implementation for individuals with different risk profiles.

## Data Availability

The original contributions presented in the study are included in the article/Supplementary Material, further inquiries can be directed to the corresponding author.
